# An analysis of the climate change effects on pesticide vapor drift from ground-based pesticide applications to cotton

**DOI:** 10.1038/s41598-023-36941-4

**Published:** 2023-06-16

**Authors:** Narayanan Kannan

**Affiliations:** Pollinator Health in Southern Crop Ecosystems Research Unit, United States Department of Agriculture-Agricultural Research Service, Stoneville, MS 38776 USA

**Keywords:** Environmental sciences, Mathematics and computing

## Abstract

Vapor drift of applied pesticides is an increasing concern. Among the major crops cultivated in the Lower Mississippi Delta (LMD), cotton receives most of the pesticides. An investigation was carried out to determine the likely changes in pesticide vapor drift (PVD) as a result of climate change that occurred during the cotton growing season in LMD. This will help to better understand the consequences and prepare for the future climate. Pesticide vapor drift is a two-step process: (a) volatilization of the applied pesticide to vapors and (b) mixing of the vapors with the atmosphere and their transport in the downwind direction. This study dealt with the volatilization part alone. Daily values of maximum and minimum air temperature, averages of relative humidity, wind speed, wet bulb depression and vapor pressure deficit for 56 years from 1959 to 2014 were used for the trend analysis. Wet bulb depression (WBD), indicative of evaporation potential, and vapor pressure deficit (VPD), indicative of the capacity of atmospheric air to accept vapors, were estimated using air temperature and relative humidity (RH). The calendar year weather dataset was trimmed to the cotton growing season based on the results of a precalibrated RZWQM for LMD. The modified Mann Kendall test, Pettitt test and Sen’s slope were included in the trend analysis suite using ‘R’. The likely changes in volatilization/PVD under climate change were estimated as (a) average qualitative change in PVD for the entire growing season and (b) quantitative changes in PVD at different pesticide application periods during the cotton growing season. Our analysis showed marginal to moderate increases in PVD during most parts of the cotton growing season as a result of climate change patterns of air temperature and RH during the cotton growing season in LMD. Estimated increased volatilization of the postemergent herbicide S-metolachlor application during the middle of July appears to be a concern in the last 20 years that exhibits climate alteration.

## Introduction

In the United States, the Federal Insecticide, Fungicide, and Rodenticide Act (FIFRA)^1^ is the federal statute that governs the registration, distribution, sale, and use of pesticides. Before a pesticide can be sold or distributed, it must be registered/licensed with the United States Environmental Protection Agency (USEPA) under FIFRA. For registering a pesticide with the USEPA, the applicant must show that using the pesticide according to the label directions will not generally cause unreasonable adverse effects to human health or the environment. To enforce FIFRA requirements, the USEPA conducts inspections, surveillance, and pesticide sampling and analysis. Assessment of inhalation exposure from pesticide volatilization is a part of the USEPA’s registration and review process. A state may regulate any registered pesticide within the state except those prohibited by the FIFRA. The state can also register additional uses of a federally registered pesticide. However, the state will not impose any requirements for labeling/packaging in addition to those imposed by FIFRA. Off-target drift is one of the adverse effects of pesticide application causing concern to the health of humans and sensitive plants and animals.

Off-target drift and any other pesticide-related environmental concerns can be reported to the state pesticide regulatory agency (for Mississippi, the Mississippi Department of Agriculture & Commerce Bureau of Plant Industry, Pesticide Program), a local agency such as a county extension office or to the USEPA directly. The other source of reporting can be the pesticide manufacturer^2^. The report should be accompanied by clear evidence such as photographs or samples of damaged foliage, logs of weather (temperature, relative humidity and wind speed), spray application details of nearby farmers, and statements from witnesses. Additionally, continuous further monitoring of the damage is highly recommended. If the regulatory agency conducting the investigation discovers a violation of regulations, it levies fines against the applicator. However, the fines paid to the agency do not provide compensation to the person(s) who reported damage. The compensation for the damages to the seeker can be obtained either by negotiation with the applicator or by means of a civil lawsuit^3^. Currently, the financial burden of funding state pesticide investigations is borne by taxpayers. To mitigate the taxpayer burden and to financially compensate the owners of the injured property, a compensation approach is proposed in a recent study^4^. Under this proposal, a state program would insure properties suffering from damage from potential pesticide applications. Every purchaser of the potential pesticide would pay a fee that would be used to fund the state program dealing with pesticide application-related problems^4^.

Off-target pesticide drift is very important because under favorable environmental conditions, pesticide vapors can be carried over longer distances^5–7^ and can cause damage to crops, animals, or humans. Drifted pesticides were detected in the atmosphere up to three times the canopy height^8^. It is important to understand pesticide drift and develop strategies to mitigate it. There are two different types of drift: (a) particle drift, which includes dust/droplets, and (b) vapor drift, which is generated by the volatilization of the pesticide during and after its application. This manuscript addresses the vapor drift resulting from volatilization.

Pesticide vapor drift can be viewed as a two-step process: (a) volatilization of the applied pesticide to vapors and (b) mixing of the vapors with the atmosphere and their transport in the downwind direction^9^. The volatilized pesticides sent to the atmosphere could return to the land surface by rain/snow and dry or wet deposition, causing off-target drift. When conditions are favorable, volatilized pesticides can be deposited on surface water^10^. The study by Taylor et al.^11^ provided some important insights into pesticide volatilization, such as (a) systemic pesticides have low volatility from plants and (b) as plants mature, increasing leaf size decreases volatilization; however, the proportion of pesticides landing on plants increases with maturity^11^. Volatilization is documented as a major dissipation pathway for some pesticides^12,13^. In the European Union, for registering a pesticide, estimation of potential volatilization of the pesticide to the air is an important prerequisite^14^. Typically, volatilization can last for approximately a few days to a few weeks after pesticide application^13^.

Pesticide volatilization is a function of both the physical and chemical characteristics of the pesticide and the environmental factors^14,15^. The vapor pressure of a pesticide is one of the most important parameters determining its volatilization. The vapor pressure of the pesticide could be treated as fugacity or escape tendency. Because of the smaller concentration of pesticide vapors in the atmosphere, we can assume that pesticide vapors will behave like any other gas^16^. The fugacity capacity of the air phase is the same for all chemicals^17^, including water vapor and pesticide vapor.

Water evaporation from pesticide-applied soil or leaf surfaces induces an upward flow of solution, which carries pesticides and other solutes^17^. Additionally, if weather parameters are favorable, pesticides can volatilize from nonwet surfaces as well. Therefore, an analysis of the evaporation potential of the pesticide and the capacity of air in the atmosphere to accept any vapor using historic long-term climatic data will shed more light on pesticide volatilization under climate change. Wet bulb depression (WBD) and vapor pressure deficit (VPD) are indicative of the abovementioned two parameters.

If the same pesticide and quantity are applied using similar application equipment to a particular crop year after year, the vapor drift is then a function of changes in weather parameters alone. Based on this idea, the goal of the study was formulated to analyze whether climate change that had already occurred in the study area had increased pesticide vapor drift. The specific objectives are (a) to collect long-term historic weather data to look at climate change patterns, (b) to put together the details of typical pesticide application operations carried out for cotton in the region, and (c) to estimate the likely changes in vapor drift for climate change conditions using easily computable parameters such as wet bulb depression (WBD) and vapor pressure deficit (VPD). There is some evidence in the literature connecting pesticide volatilization with WBD^18^ and VPD^19^; therefore, the use of WBD and VPD is a feasible way to estimate pesticide volatilization under climate change.

The study area chosen is a part of the Lower Mississippi Delta (LMD), an intensively cultivated and one of the most productive agricultural regions in the US. Corn, soybean, and cotton are the most widely cultivated crops in the region. The majority of pesticide use in the LMD region is for cotton. Although cotton is not the largest user of pesticides worldwide, it is estimated that more than 80% of the global cotton crop could be lost to different pests without using pesticides in the production system^20^. This finding outlines the need for the use of pesticides for cotton production. Therefore, pesticide applications during cotton production in the LMD were chosen for the vapor drift analysis.

The uniqueness of the present study is the qualitative and quantitative estimation of pesticide vapor drift using simple and easy-to-calculate weather-based parameters such as wet bulb depression and vapor pressure deficit.

## Methods

### Study area

**Figure 1:**
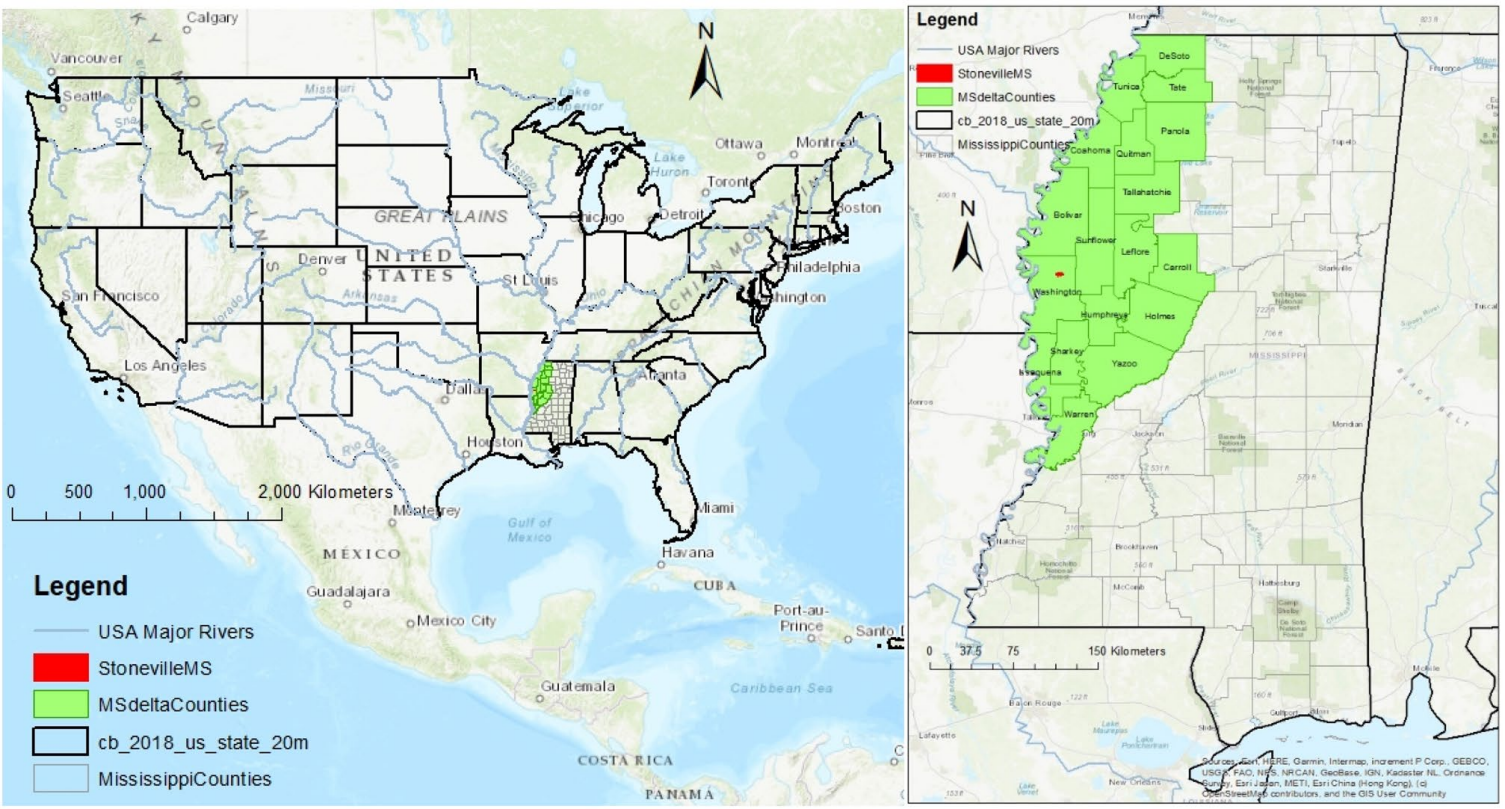
Location of the study area.

The study was conducted in one of the important cotton production regions in the country located in Stoneville, MS, a part of Washington County and the LMD region (Fig. 1). Although the area receives plenty of precipitation, some irrigation is required to supplement highly variable precipitation events during crop growth. Fertile soil (Dubbs silt loam: (fine-silty, mixed, thermic Typic Hapludalfs)) and adequate sunshine generally support successful crop production. Soybeans, cotton, and corn are the major crops in the region. However, other crops are also cultivated. The majority of pesticide use in the region is for cotton. Therefore, pesticide applications during cotton production were chosen for analysis. This study was focused on understanding how climate change occurred in the region and how it could have impacted pesticide vapor drift in the region; therefore, this work is unique from many other studies.

### Data used

Daily observations of maximum and minimum air temperatures (or dry bulb temperature), wind speed (horizontal component (u) only), and relative humidity were used for the period 1960–2014. The weather data were obtained from the weather station located at Stoneville, MS, and the Delta Research and Extension Center, Mississippi State University^21^. For the same duration, the length of the growing season (May first week to October second week) was obtained from the Root Zone Water Quality Model (RZWQM2)^22–24^. Based on the length of the cotton growing season reported by the model each year, the weather observations were trimmed to represent the cotton growing period only for a meaningful analysis.

### Estimation of wet bulb depression (WBD)

In this study, a simple weather-based parameter, namely, wet bulb depression, which is indicative of evaporation potential, is used to analyze the potential changes in pesticide vapor drift arising from the climate change that occurred in the region. Wet bulb depression is the difference between the observed air temperature (or dry bulb temperature) and the wet bulb temperature. The wet bulb temperature was estimated from the dry bulb temperature and relative humidity using the Stull formula (Eq. 2) as described by the omni-calculator^25^. Equation (2) is applicable for relative humidity between 5 and 99% and temperatures between − 20 and 50 °C.

1 $$WBD=T-{T}_{w}$$

where T is the dry bulb temperature (°C) of air (or air temperature) and T_w_ is the wet bulb temperature (°C).

2 $$\begin{aligned} {T}_{w} & =T\times {\mathrm{tan}}^{-1}[0.151977\times \surd (rh+8.313659)]+{\mathrm{tan}}^{-1}(T+rh)-{\mathrm{tan}}^{-1}(rh-1.676331) \\ & \quad +[(0.00391838\times {rh}^\frac{3}{2})\times {\mathrm{tan}}^{-1}(0.023101\times rh)]-4.686035 \end{aligned}$$

where T_w_ is the wet bulb temperature (°C), T is the dry bulb temperature (normal air temperature) (°C), rh is relative humidity (%).

### Estimation of vapor pressure deficit (VPD)

The vapor pressure deficit between the evaporating surface and air indicates the capacity of the space available in the air for accepting vapors from the evaporating surface. In the case of pesticides applied to soil/plant, more VPD is indicative of more volatilization from the applied surface. VPD is estimated based on the following equations:

3 $$VPD={VP}_{EvapSurf}-{VP}_{air}$$

where VP_EvapSurf_ is the vapor pressure of the evaporating surface (leaf/soil) in kPa, VP_air_ is the vapor pressure of air in kPa.

Vapor pressure can be estimated using the Tetens^26,27^ equation (Eq. 3):

4 $${VP}_{EvapSurf}=0.61708\times {e}^{\frac{(17.27\times T)}{(T+237.3)}}$$

where T is the temperature of the evaporating surface in °C. For our analysis, long-term records of leaf/soil temperatures are not available. Therefore, air temperature is used for computations. However, in general, plants without water stress will have leaf temperatures cooler than the air temperature. Because we use air temperature in Eq. (3), the result is the same as the saturation vapor pressure of air (VP_SatAir_). The vapor pressure (VP) of air can be estimated from Eq. (5) as follows:

5 $${VP}_{air}={VP}_{SatAir}\times RH$$

where RH is the relative humidity of air in %.

### Trend analysis

As a part of the trend analysis, the modified Mann–Kendall test^28^, Pettitt test^29^, and Sen’s slope^30^ were performed using R software for each time series. All the tests were carried out at a level of significance less than 5% probability (p ≤ 0.05). Together, Mann–Kendall and Sen’s slope tests have been used in several hydro climatological studies^31–33^. The growing season average of wet bulb depression and vapor pressure deficit VPD (one average value for the growing season in the calendar year) and averages of temperature, relative humidity, wind speed and wet bulb depression and VPD at critical pesticide application stages for cotton (Table 1) were used.

**Table 1:** Timing and type of chemical application for cotton grown in Stoneville, MS (the window of dates is selected based on three years of field data to accommodate year-to-year changes in dates of chemical application).

Timing of operation		Proportion of (pesticide + adjuvant): water	Chemical application	Name of the active ingredients
From	To			
13-May	19-May	0.50: 99.50	Preemergence herbicide	Fluometuron: 1,1-dimethyl-3-(a,a,a-trifluoro-m-tolyl) urea, pendimethalin: N-(1-ethylpropyl)-3,4-dimethyl-2,6-dinitrobenzenamine
14-Jun	20-Jun	0.15: 99.85	Post-emergence herbicide	Glufosinate-ammonium
28-Jun	4-Jul	0.55: 99.45	Insecticide and growth regulator application along with an adjuvant	Imidacloprid: 1-[(6-Chloro-3-pyridinyl)methyl]-/tf-nitro-2-imidazolidinimine, Novaluron: 1-[3-chloro-4-(1,1,2-trifluoro-2-trifluoro-methoxyethoxy)phenyl]-3-(2,6-diflurobenzoyl) urea, Mepiquat Chloride: N,N-dimethylpiperidinium chloride, 3-oxapentane-1,5-diol, propane-1,2,3-triol, alkylphenol ethoxylate, polydimethylsiloxane
12-Jul	18-Jul	0.17: 99.83	Post-emergence herbicide application	Glufosinate-ammonium, S-metolachlor
13-Jul	19-Jul	0.50: 99.50	Insecticide application	Sulfoxaflor, Mepiquat Chloride: N,N-dimethylpiperidinium chloride, 3-oxapentane-1,5-diol, propane-1,2,3-triol, alkylphenol ethoxylate, polydimethylsiloxane
23-Jul	29-Jul	0.10: 99.90	Insecticide	Lambda-cyhalothrin, Chlorantraniliprole
5-Aug	11-Aug	1.22: 98.78	Insecticide along with an adjuvant	Acephate, Lambda-cyhalothrin, Chlorantraniliprole, Mepiquat Chloride: N,N-dimethylpiperidinium chloride, 3-oxapentane-1,5-diol, propane-1,2,3-triol, alkylphenol ethoxylate, polydimethylsiloxane
12-Sep	27-Sep	0.04: 99.96	Defoliant	Thidiazuron: N-phenyl-N'-1, 2,3-thidiazol-S-ylurea, Diuron: 3-(3,4-dichlorophenyl)-1,1-dimethylurea
29-Sep	5-Oct	0.60: 99.40	Boll busting/growth regulation	55.4–Ethephon (2-chloroethyl) phosphoni acid

It should be noted that there were nine distinct pesticide application periods identified for cotton cultivation in the study area. However, periods four and five were only one day before/after (Table 1). Therefore, from the perspective of baseline vs. climate change analysis, periods four and five were combined, which resulted in eight periods for trend analysis results. The pesticide application details described in the manuscript are based on a summary of the commonly used land management practices for cotton in the region. We have not conducted any field experiment(s) as a part of this study.

#### Modified Mann–Kendall test

The Mann–Kendall test is a nonparametric test to investigate the existence of any monotonous increasing or decreasing trends in data. Most weather data will have autocorrelation (dependency of one data entry on the previous one, for example, dependence of today’s relative humidity on the previous day’s relative humidity). This type of autocorrelation might interfere with the clear identification of trends in the data. Therefore, the modified Mann–Kendall test that addresses the autocorrelation in the data were used in this study. The modified Mann–Kendall test was performed using the package ‘modifiedmk’ developed for use in R.

#### Pettitt test

The nonparametric Pettitt test is used to identify the single change point in the continuous time series of data. The Pettitt test is based on the rank-based Mann–Whitney test^34^. It has been used in several climatological studies^35–37^. The ‘trend’ package available in R was used to perform the Pettitt test.

#### Sen’s slope

Sen’s slope test is used to estimate the magnitude of the trend (linear rate of change) in the data. Both the slope and the intercept are computed as a part of this test. Sen’s slope is a median parameter estimated from the series of linear slopes estimated between sets of two data points. The test provides 95% confidence limits of results as well. It was performed using the package ‘trend’ in R.

#### Hypotheses tested

The null hypotheses (H_0_) tested were (a) there is no monotonic trend in the time series, (b) there is no change point in the time series and (c) there is no change in the magnitude of the trend in the time series for the modified Mann–Kendall test, Pettitt test and Sen’s slope test, respectively. The alternate hypotheses (H_A_) were (a) there is a monotonic trend in the time series, (b) a change point exists in the time series and (c) there is a change in the magnitude of the trend in the time series for the modified Mann–Kendall test, Pettitt test and Sen’s slope test, respectively.

#### How are the results analyzed?

To analyze the results, statistical significance was used. Statistical significance indicates whether the results obtained are very likely or could have been obtained by chance. In the context of trend analysis, it measures the probability of the null hypothesis becoming true with a predefined significance level. In this study, a 5% significance level is used to judge the results. For example, suppose the results show a decreasing trend in relative humidity and are statistically significant; in that case, the relative humidity declines estimated by trend analysis is very likely, and the results are statistically defensible. If the probability values are relatively higher than the 5% level, the results are less reliable, and there is uncertainty around the results. In the manuscript, this item is categorized as less significant. If the trend analysis results are not statistically significant, it indicates that the result could have occurred by chance, and they are not reliable.

## Results

### Changes in seasonal average weather parameters

The trend analysis results of the magnitude of the seasonal average and extreme parameters are presented in Table 2. The corresponding statistical significance of the results is presented in Table 3. There is a monotonous increase in the seasonal average air temperature. The significant change point corresponds to 1992. With respect to the baseline period (1960–1992) average temperature (25.0 °C (77.0 °F)), there was an increase of 1.1 °C (1.9 °F) in the altered (1993–2014) period (26.1 °C (78.9 °F)). The rate of increase is 0.2 °C/decade (or 0.35 °F/decade) (Table 2). Similarly, there is an increase in relative humidity and an increase in wind speed, the details of which are presented in Table 2. All the trend analysis results are statistically significant for average temperature, average relative humidity, and minimum temperature. None of the trend analysis results are significant for minimum wind speed. For all the other parameters, some tests showed statistical significance, and others showed no significance (Table 3).

**Table 2:** Trend analysis results on the magnitude of seasonal average, minimum and maximum weather parameters during the cotton growing season.

Growing season parameter	Monotonous trend	Change point year	Absolute change	Rate of change/decade
Average temperature	Increase	1992	1.1 °C (1.9 °F)	0.2 °C (0.35 °F)
Average relative humidity	Increase	1969	2.2%	0.25%
Average wind speed	Increase	1970	0.11 m/s (0.24 mph)	0.01 m/s (0.02 mph)
Minimum temperature	Increase	1985	2.1 °C (3.8 °F)	0.45 °C (0.81 °F)
Minimum wind speed	Decrease	1973	0.07 m/s (0.15 mph)	0.0 m/s (0.0 mph)
Maximum temperature	Increase	1996	1.3 °C (2.3 °F)	0.23 °C (0.41 °F)
Maximum wind speed	Increase	1979	0.34 m/s (0.72 mph)	0.06 m/s (0.13mph)
Average wet bulb depression	Decrease	1970	0.26 °C (0.5 °F)	0.05 °C
Average vapor pressure deficit	Increase	2004	0.1 kPa	0.004 kPa

**Table 3:** Statistical significance of trend analysis results on seasonal average and extreme weather parameters (Zc, Z, and U are test statistic values for the modified Mann–Kendall, Pettitt, and Sen’s slope tests, respectively).

Seasonal parameter	Test statistic	Test statistic value		
		Mann–Kendall	Pettitt	Sen’s slope
Average temperature	Z_c_/Z/U	4.55	395	2.42
	p	5.28 × 10^–6^	7.96 × 10^–3^	0.015
	τ	0.225	NA	NA
	Statistical significance	Significant	Significant	Significant
Average relative humidity	Z_c_/Z/U	3.62	305	2.24
	p	2.94 × 10^–4^	0.074	0.025
	τ	0.208	NA	NA
	Statistical significance	Significant	Less Significant	Significant
Average wind speed	Z_c_/Z/U	1.73	237	1.15
	p	0.083	0.273	0.251
	τ	0.107	NA	NA
	Statistical significance	Less significant	Not significant	Not significant
Minimum temperature	Z_c_/Z/U	5.93	344	2.19
	p	2.96 × 10^–9^	0.03	0.028
	τ	0.203	NA	NA
	Statistical significance	Significant	Significant	Significant
Minimum wind speed	Z_c_/Z/U	− 0.66	271	− 0.42
	p	0.506	0.148	0.678
	τ	− 0.039	NA	NA
	Statistical significance	Not significant	Not significant	Not Significant
Maximum temperature	Z_c_/Z/U	3.04	298	1.68
	p	2.33 × 10^–3^	0.086	0.093
	τ	0.155	NA	NA
	Statistical significance	Significant	Less significant	Less significant
Maximum wind speed	Z_c_/Z/U	3.86	212	1.19
	p	1.15 × 10^–4^	0.407	0.234
	τ	0.111	NA	NA
	Statistical significance	Significant	Not significant	Not significant
Average wet bulb depression	Z_c_/Z/U	− 2.63	251	− 1.704
	p	8.4 × 10^–3^	0.215	0.088
	τ	− 0.157	NA	NA
	Statistical significance	Significant	Not significant	Less significant
Average vapor pressure deficit	Z_c_/Z/U	− 0.72	218	− 0.48
	p	0.49	0.37	0.63
	τ	− 0.045	NA	NA
	Statistical significance	Not Significant	Not significant	Not significant

### Qualitative changes in pesticide vapor drift

**Figure 2:**
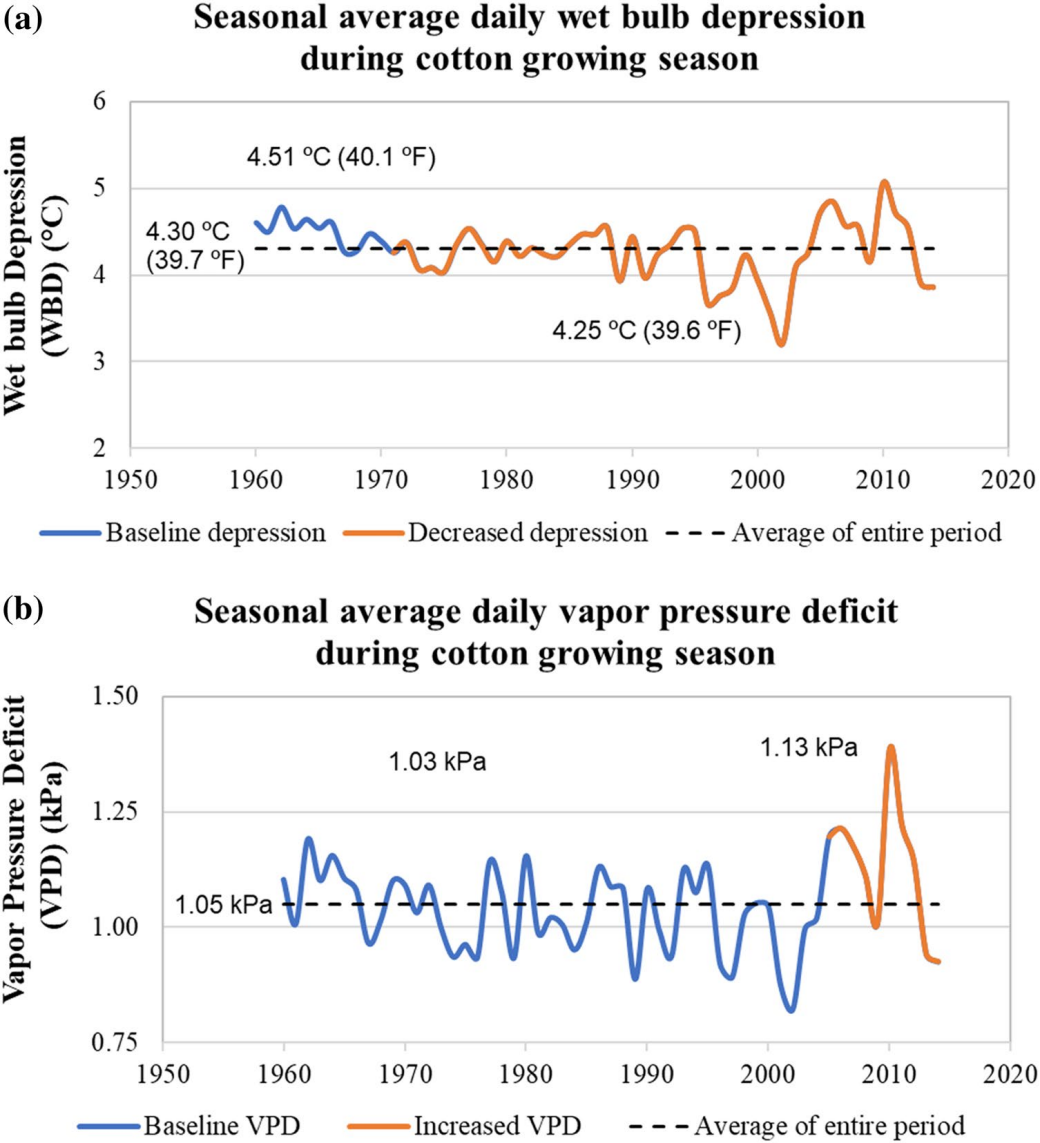
(**a**) Monotonic trends in evaporation potential as indicated by wet bulb depression. (**b**) Monotonic trends in the capacity of space for vapors in air as indicated by vapor pressure deficit.

Trends in wet bulb depression (estimated from the air temperature and relative humidity) indicative of evaporative demand and vapor pressure deficit indicative of room in air for pesticide vapors are shown in Fig. 2. Decreasing trends are noticed for the evaporative demand, and the results are statistically significant for the modified Mann–Kendall test, less significant for Sen’s slope and not significant for the Pettitt test. Conversely, increasing trends are observed for VPD, although the numerical statistics indicate otherwise. However, none of the VPD results are statistically significant.

The volatilization of the applied pesticide is a function of the pesticide, atomization pattern, and weather parameters. Given the scenario of the same chemical application using similar atomization methods each year, any changes in weather parameters will likely alter the volatilization of the particular chemical. The decrease in evaporative demand (as shown in Fig. 2a) indicates decreased (climate change-induced) volatilization of the pesticide in the LMD. The increased VPD indicates a higher possibility for volatilization, contradicting the results of WBD or evaporative demand. Although both VPD and WBD use both the temperature and relative humidity of air, there are some differences in the way they are mathematically used to derive the respective parameters. VPD shows the dryness of air or the capacity to accept additional vapors whereas WBD is influenced by humidity. Depending on atmospheric conditions, both WBD and VPD can provide different insights into the evaporative conditions of the atmosphere. Moreover, the information provided in Fig. 2 is based on seasonal averages alone. Analyzing similar details on each pesticide application period using historic weather data will shed more light on climate-induced pesticide volatilization patterns.

### Climate change during critical pesticide application periods within the cotton growing season

**Figure 3:**
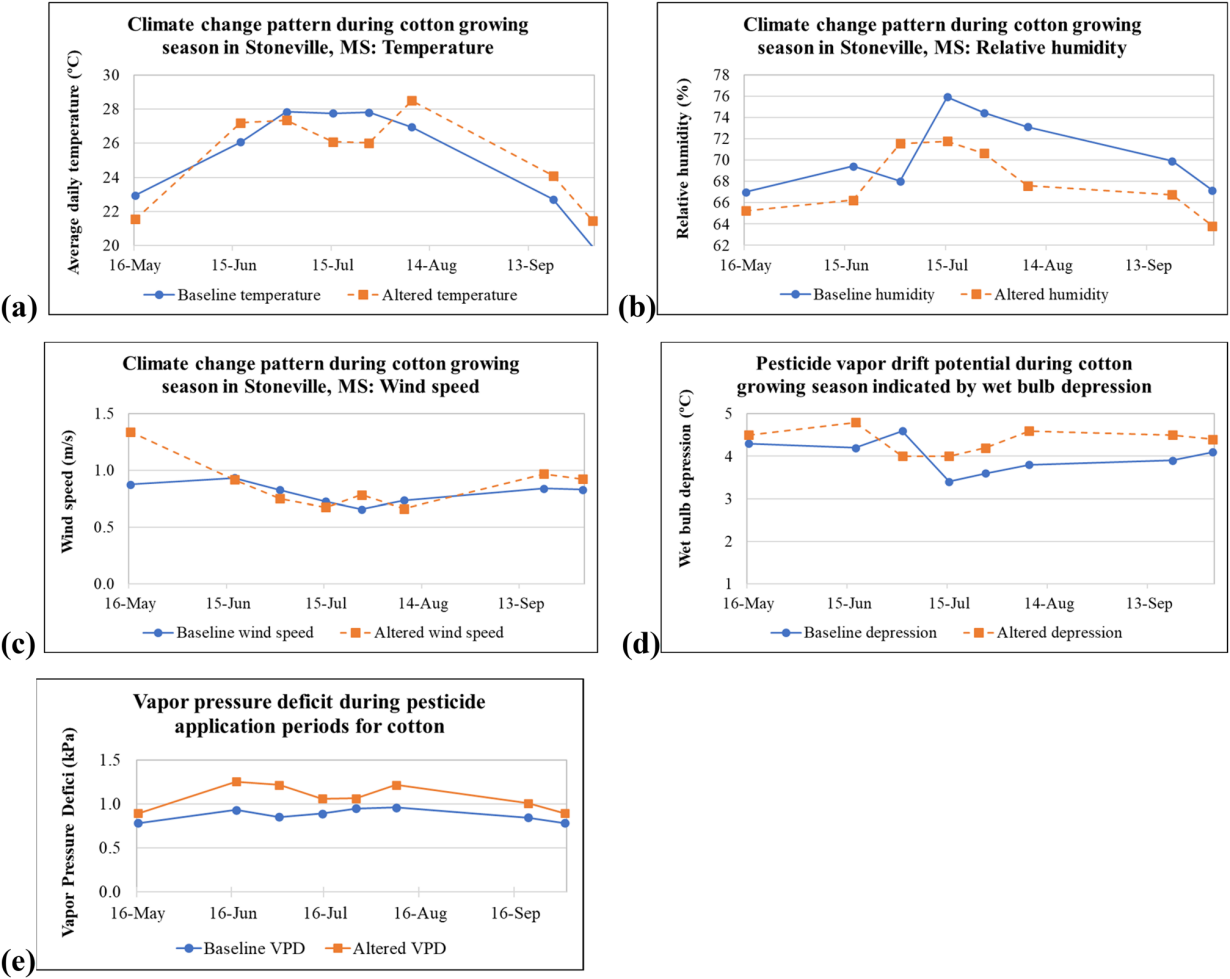
Details of the trends in weather parameters during different stages of cotton growth (**a**) air temperature, (**b**) relative humidity, (**c**) wind speed, (**d**) wet bulb depression, (**e**) vapor pressure deficit.

**Figure 4:**
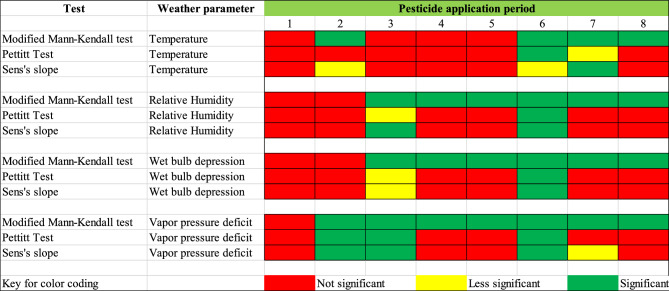
Summary of trend analysis results for temperature, relative humidity, and wind speed.

The trend analysis results of weather parameters at important stages of pesticide application during cotton growth are presented in Fig. 3 and Table 4. The corresponding statistical significance of the results is presented in Fig. 4. During the preemergent herbicide application and the two insecticide applications during the beginning of summer, there was a temperature decline (Fig. 3a) for the climate change scenario when compared to the baseline. However, during late spring and most of the later part of summer, there is a consistent increase in air temperature during pesticide applications for cotton in the climate change scenario. Unlike temperature, there was a decline in RH throughout the growing season between baseline and altered conditions, except during the third pesticide application period. The difference in RH between the baseline and altered climate conditions was as much as 5% for pesticide application during the later part of the summer. Although the seasonal average and seasonal extreme wind speed data showed some trends, within-season values did not show any noticeable trend except for the preemergent herbicide application (first pesticide application of the season). The trend analysis results of weather parameters are most significant for relative humidity, less significant for temperature and almost insignificant for wind speed (Fig. 4).

**Table 4:** Trend analysis results on the magnitude of average weather parameters during critical herbicide, insecticide, and growth regulator application periods.

Pesticide application period: weather parameter	Monotonous trend	Change point year	Absolute change	Rate of change/decade
Period 1: temperature	Decrease	2001	1.4 °C (2.5 °F)	0.09 °C (0.16 °F)
Period 2: temperature	Increase	1989	1.1 °C (2.0 °F)	0.28 °C (0.51 °F)
Period 3: temperature	Decrease	1980	0.5 °C (0.9 °F)	0.0 °C (0.0 °F)
Period 4: temperature	Decrease	2011	1.7 °C (3.0 °F)	0.0 °C (0.0 °F)
Period 5: temperature	Decrease	2012	1.8 °C (3.2 °F)	0.0 °C (0.0 °F)
Period 6: temperature	Increase	1997	1.5 °C (2.8 °F)	0.27 °C (1.87 °F)
Period 7: temperature	Increase	1996	1.4 °C (2.5 °F)	0.30 °C (0.53 °F)
Period 8: temperature	Increase	1993	1.5 °C (2.7 °F)	0.33 °C (0.59 °F)
Period 1: wind speed	Increase	1970	0.5 m/s (1.0 mph)	0.0108 m/s (0.02 mph)
Period 2: wind speed	Decrease	1984	0.02 m/s (0.04 mph)	0.014 m/s (0.03 mph)
Period 3: wind speed	Decrease	1999	0.08 m/s (0.16 mph)	0.00 m/s (0.00 mph)
Period 4: wind speed	Decrease	1994	0.05 m/s (0.11 mph)	0.007 m/s (0.13 mph)
Period 5: wind speed	Increase	1999	0.13 m/s (0.28 mph)	0.00 m/s (0.00 mph)
Period 6: wind speed	Decrease	1973	0.07 m/s (0.16 mph)	0.003 m/s (0.015 mph)
Period 7: wind speed	Increase	1998	0.13 m/s (0.28 mph)	0.0108 m/s (0.02 mph)
Period 8: wind speed	Increase	1984	0.09 m/s (0.20 mph)	0.03 m/s (0.06 mph)
Period 1: relative humidity	Decrease	1996	1.7%	0.09%
Period 2: relative humidity	Decrease	2004	3.2%	0.03%
Period 3: relative humidity	Increase	1980	3.6%	0.86%
Period 4: relative humidity	Decrease	2002	4.2%	0.52%
Period 5: relative humidity	Decrease	2002	3.9%	0.58%
Period 6: relative humidity	Decrease	2002	5.5%	1.08%
Period 7: relative humidity	Decrease	2002	3.1%	0.57%
Period 8: relative humidity	Decrease	2002	3.4%	0.30%
Period 1: wet bulb depression	Increase	1996	0.2 °C	0.00 °C
Period 2: wet bulb depression	Increase	2004	0.6 °C	0.00 °C
Period 3: wet bulb depression	Decrease	1980	0.6 °C	0.13 °C
Period 4: wet bulb depression	Increase	2002	0.6 °C	0.09 °C
Period 5: wet bulb depression	Increase	2002	0.6 °C	0.09 °C
Period 6: wet bulb depression	Increase	2001	0.8 °C	0.17 °C
Period 7: wet bulb depression	Increase	2002	0.6 °C	0.10 °C
Period 8: wet bulb depression	Increase	1993	0.3 °C	0.08 °C
Period 1: vapor pressure deficit	Increase	1987	0.09 kPa	Insignificant
Period 2: vapor pressure deficit	Increase	2004	0.32 kPa	0.050 kPa
Period 3: vapor pressure deficit	Increase	2004	0.36 kPa	0.038 kPa
Period 4: vapor pressure deficit	Increase	2002	0.17 kPa	0.023 kPa
Period 5: vapor pressure deficit	Increase	1996	0.11 kPa	0.028 kPa
Period 6: vapor pressure deficit	Increase	1998	0.26 kPa	0.054 kPa
Period 7: vapor pressure deficit	Increase	2002	0.16 kPa	0.028 kPa
Period 8: vapor pressure deficit	Increase	1993	0.11 kPa	0.031 kPa

Therefore, the cotton-applied pesticide vapor drift pertaining to climate change in LMD is limited to the combined effect of changes in temperature (most increases and sometimes declines) and relative humidity alone, which is shown in Fig. 3d,e. Given the same chemical and application rates using the same equipment year after year, climate change that occurred in the past in the LMD could have increased the pesticide vapor drift/pesticide volatilization during most of the cotton growing season (except application period three), as evidenced by Fig. 3d showing wet bulb depressions. In terms of proportional increases (in climate change period than baseline), the wet bulb depressions range from − 13 to 21%. However, Fig. 3e shows consistent increases in VPD, suggesting increases in vapor drift/pesticide volatilization throughout the cotton growing season. The increased proportions during the climate-altered period range from 12 to 43%.

## Discussion

Increasing air temperature during the cotton growing season has been highlighted in several previous studies^38,39^. Similarly, changes in relative humidity during the crop growth period are also documented^40^. The volatilization of applied pesticides is a function of the physico-chemical characteristics (especially vapor pressure) of pesticides^13^, plant ability to absorb chemicals, soil pH^41^, application equipment (the way pesticides are atomized)^18^ and weather parameters^13^, especially air temperature, relative humidity, and wind speed^7^. Leaf area, proportion of pesticide intercepted by canopy, and leaf height were also reported to affect pesticide volatilization^11^. Similar to this study, higher pesticide volatilization under climate change is reported in Delcour et al.^42^ and Tudi et al.^43^. Similar to this study, the analysis by Ferraro and de Paula^18^ describes wet bulb depression as one of the parameters determining pesticide volatilization. In this study, both the wet bulb depression and VPD were used to arrive at conclusions on possible climate change effects on pesticide volatilization. There are, however, differences between the results of WBD and VPD. Based on the physico-chemical characteristics of the pesticides applied for cotton in LMD (Table 5), especially the vapor pressure and Henry’s law constant^44^, it appears that pendimethalin applied in period 1 and S-metolachlor applied during period 4 show more volatility than other pesticides. The increasing trends indicated by both WBD and VPD during period 1 are statistically insignificant. Therefore, pendimethalin applied during period 1 may not have volatilized more because of climate change. However, period 4 pesticide application corresponding to the S-metolachlor application shows increasing trends both by WBD and by VPD (Table 4, Figs. 3 and 4) that are statistically significant. Therefore, S-metolachlor applied in cotton during application period 4 could have volatilized more in LMD than any other pesticide on the list. It should be noted that this conclusion is based on the physico-chemical properties of pesticides and trend analysis of weather and weather-derived parameters. Field data collection and analysis are needed to verify this result.

**Table 5:** Important physico-chemical properties of the pesticides used in cotton cultivation.

Pesticide active ingredient	Molecular mass	Solubility in water at 20 °C (mg/L)	Density (g/ML)	Vapor pressure (mPa)	Henry’s law constant at 25 °C (Pa m^3^ mol^−1^)
Fluometuron	232.2	111.00	1.39	0.12	2.63 × 10^–4^
Pendimethalin	281.3	0.33	1.17	3.34	1.27
Glufosinate-ammonium	198.2	500,000.0	1.32	3.10 × 10^–2^	4.48 × 10^–9^
S-Metolachlor	283.8	40.0	1.12	3.7	2.20 × 10^–3^
Thidiazuron	220.2	20.0	1.51	3.00 × 10^–6^	3.30 × 10^–8^
Mepiquat chloride	149.7	747,000	1.16	1.00 × 10^–5^	2.99 × 10^–12^
Imidacloprid	255.6	610	1.54	4 × 10^–7^	1.65 × 10^–10^
Acephate	183.2	818,000	1.35	2 × 10^–10^	5 × 10^–8^
Chlorantraniliprole	483.1	0.88	1.51	6.3 × 10^–09^	3.2 × 10^–09^
Lambda-cyhalothrin	449.8	0.005	1.33	0.002	2.00 × 10^–02^
Polydimethylsiloxane	74.0	Insoluble	1.00	6.67 × 10^–4^	NA

Given below are the sources of the information presented in the table.

Pesticide Properties Database (PPDB), University of Hertfordshire, UK^45,46^.

Apart from climate change, other factors could be responsible for the increased volatilization of pesticides. Higher pesticide volatilization from moist soils than dry soils is documented by Schneider and Goss^47^. A dry soil will have plenty of surface area for the pesticide to adsorb. However, rainfall or irrigation before or after application will significantly reduce the surface available for the pesticide to adsorb and become readily available for volatilization^47^. This could also be viewed from another dimension of timing decisions on pesticide application. For example, targeting the pesticide application date prior to a few dry days and avoiding times with high heat will help to minimize volatilization problems. This can be accomplished by having access to a reliable weather forecast for a week. Other factors, such as human decisions on spraying equipment selection (type and number of nozzles), application method (aerial vs. ground), and respecting or not respecting the application instructions recommended in the pesticide label, could also affect pesticide volatilization. Ensuring the compliance of the other factors mentioned here is a prerequisite to pinpointing the role of climate change alone in increasing pesticide volatilization and vapor drift.

### Limitations of the study

To interpret the results, wet bulb depression, which is a function of temperature and relative humidity, was used in this study. Although wind speed is certainly a factor influencing volatilization, it was not included in the analysis because the extent of changes in wind speed during the climate change scenario (when compared to baseline) is negligible and the trends were not statistically significant. However, subdaily or instantaneous wind speeds for different scenarios could have provided some useful information. However, they were not available. This could have some errors associated with the analysis results. To estimate the vapor pressure of the pesticide evaporating surface (leaf/soil), air temperature is used instead of leaf/soil temperature. For our analysis, long-term records of leaf/soil temperatures are not available. Therefore, air temperature is used for computations. However, in general, plants without water stress will have leaf temperatures cooler than the air temperature. Additionally, soil temperatures vary from air temperature. These could have introduced errors in the results. This study attempted to quantify only the volatilization part of the vapor drift. The other important part of vertical mixing of the evaporated pesticide vapors in the atmosphere, its degradation in the atmosphere and the subsequent transport in the downwind direction are not addressed. However, they could be pursued as a future effort.

## Summary and conclusions

In this study, an attempt was made to ascertain the possible consequences of climate change on pesticide vapor drift using data on pesticide applications to cotton in Stoneville, MS, USA. Pesticide vapor drift is a two-step process: (a) volatilization of the applied pesticide to vapors and (b) mixing of the vapors with the atmosphere and their transport in the downwind direction. This study dealt with the volatilization part alone. Fifty-five years of daily weather data from 1960 to 2014 were used for the trend analysis. Patterns in wet bulb depression (WBD) and vapor pressure deficit (VPD) estimated from weather data were related to pesticide volatilization potential. Based on the results obtained, we can conclude the following:

- Significant changes occurred in the seasonal average daily temperature and seasonal average relative humidity.
- Noticeable changes occurred in daily average temperature, and significant changes were noticeable in the daily average relative humidity during critical stages of pesticide application for cotton.
- Based on the physico-chemical characteristics of pesticides and trend analysis results of weather, it appears that the S-metolachlor applied in cotton during the middle of July could have volatilized more in the study area than any other pesticide.
- In summary, the changes in temperature and declines in relative humidity during the cotton growing season could have increased the pesticide volatilization/vapor drift estimated from WBD and VPD in the region.

## Data Availability

Data used in the study are available on request.
